# Uncovering the Evidence for Sustainability in Urology: A Scoping Review

**DOI:** 10.5152/tud.2024.24093

**Published:** 2024-05-01

**Authors:** Habeeb Abdulrasheed, Ayokunle Adenipekun, Waleed Elsayed, Mohamed S. Mohsin, Daniel Madarshahian, Humood Almedej, Banan Osman

**Affiliations:** Department of Urology, Heartland Hospital, University Hospitals Birmingham NHS Foundation Trust, Birmingham, United Kingdom

**Keywords:** Carbon footprint, greenhouse gas emissions, low-emission anaesthetics, reusable urology devices, single-use urology devices, sustainability in healthcare, urology services

## Abstract

**Objective:**

This article focuses on the environmental impact of urology devices and procedures in hospitals and identifies practices that can reduce greenhouse gas emissions associated with urology services.

**Materials and Methods:**

A scoping review following the Preferred Reporting Items for Systematic Reviews and Meta-Analyses (PRISMA) guidelines was conducted using MEDLINE, EMBASE, and Google Scholar to find studies on the carbon footprint of urologic procedures and sustainable practices.

**Results:**

We identified 14 studies, 6 of which used life cycle assessments to compare the environmental impact of single-use and reusable urology devices. Three studies favored single-use devices, 2 favored reusable ones, and 1 found no significant difference, with the sterilization of reusable devices being a major carbon contributor. To enhance sustainability in urology, 8 articles suggested measures including day-case procedures, minimizing low-value care, drapeless cystoscopy, fluid management systems, using quick response (QR) codes in documentation, telehealth initiatives, and low-emission anesthetics.

**Conclusion:**

Promoting sustainability in healthcare requires more than just using reusable equipment; it necessitates a comprehensive approach from manufacturing to disposal, including the carbon footprint of sterilization. Encouraging low-emission anesthetics, QR codes, and telemedicine can significantly reduce emissions in urology.

**Main Points**

- Sustainability in healthcare requires addressing not only the adoption of reusable equipment but also the carbon footprint associated with its sterilization.
- Anesthetists are advised to embrace low-emission anesthetics.
- Centralizing urology services, the adoption of telemedicine, and one-stop urology clinics may play crucial role in lowering the carbon footprint from urology practice.
- Future research should prioritize assessing the environmental impact of suggested initiatives for urology sustainability, such as utilizing barcodes in medical documentation.

## Introduction

The global healthcare sector is a significant contributor to carbon emissions and environmental pollution.^1^ If emissions from the global healthcare industry are compared with those of individual nations, healthcare would be the fifth largest producer of greenhouse gases (GHGs) in the world. Overall, the healthcare sector’s carbon footprint accounts for about 5% of global net emissions.^2^

The National Health Service of the United Kingdom (NHS UK) contributes 24.9 million tonnes of carbon dioxide (CO_2_) and 4% of national greenhouse gas emissions (GHGe).^3^ The United States health sector, on the other hand, contributes toward 10% of the national GHG emissions and 655 million tonnes of CO_2_ equivalent per annum.^4^

The operating room is the largest contributor to GHG emissions within hospitals and accounts for up to 28% of hospital waste while consuming more energy than any other area within the hospital, primarily due to heating, ventilation, and air conditioning.^5^ In the NHS, the supply chain contributes 62% of the carbon footprint, with 24% coming from healthcare delivery and 10% from staff and patient travel.^6^ These figures have prompted the NHS to launch a campaign to decarbonize the system and achieve a net-zero carbon footprint by 2050.^7^

In addition to depleting the ozone layer, carbon emissions from healthcare delivery also contribute to air pollution, the release of carcinogens into the environment, water scarcity, and ecotoxicity.^3^ Environmental consciousness among medical staff and the public has a focus on sustainability as well as investments toward promoting sustainable practice. Sustainability entails meeting present needs without endangering future resources.^8^ In healthcare, sustainable surgery involves transitioning surgical practices to alternative models of care that generate less waste.^9^

Urology stands out due to its resource-intensive nature, making it crucial to implement efforts to diminish carbon footprints. Urology services contribute to this footprint through various means, including multiple non-reusable scopes, guidewires, baskets, irrigation fluids and other consumables, operating room procedures, general and local anesthetics, investigations, and the transportation of patients and staff to hospitals.^10^

Sustainability in urology, therefore, encompasses the adoption of eco-friendly and socially acceptable practices aimed at minimizing the adverse impacts of urological services while upholding ethical standards and the quality of patient care.^8^ Implementing changes in practice, particularly within the operating theater, involves actions like recycling waste, utilizing reusable devices, and advocating more for local anesthesia where applicable, to reduce clinical waste and carbon emissions from urology services. Additionally, it necessitates cutting down on unnecessary investigations and transportation frequencies.^9^ The objective of this review is to assess the available literature concerning sustainability in urology, focusing on aspects such as the carbon footprint of urologic procedures and practices aimed at mitigating the GHG emissions associated with urology services.

## Material and Methods

### Eligibility Criteria

We included all studies that provided data on the environmental impact of urology practice or sustainability in urology regardless of the period of publication. Excluded from our review were review articles, meta-analyses, commentaries, case reports, letters to the editors, and studies published in languages other than English.

### Information Sources, Search, Selection and Data Charting

Our review was carried out using the Preferred Reporting Items for Systematic Reviews and Meta-Analyses (PRISMA) checklist. PubMed, EMBASE, and Google Scholar were searched to identify all relevant articles. The latest literature search was performed on 27 March 2024. MeSH words used to search were “Sustainability” and “Urology,” “Environmental sustainability” and “Urology,” and the term “Greener Urology.” The search was carried out independently by the authors, and search results were imported into Rayyan.ai, a systematic review software, for screening and exclusion of duplicates. Articles were selected based on their relevance to the topic.

### Quality Appraisal of Data Sources and Result Synthesis

After the keyword search, study titles and abstracts were screened, prioritizing articles on sustainability in urology. Full texts were obtained for the relevant articles and inclusion and exclusion criteria were applied. The references of included studies were screened for studies not identified through the original search and included. All discrepancies were resolved by mutual consensus among the authors.

The following information regarding each eligible study was recorded: authors, country of origin of the first author, journal of publication, year of publication, the objective of the study, and the main findings or recommendation.

## Results

### PRISMA Flowchart

**Figure 1.**
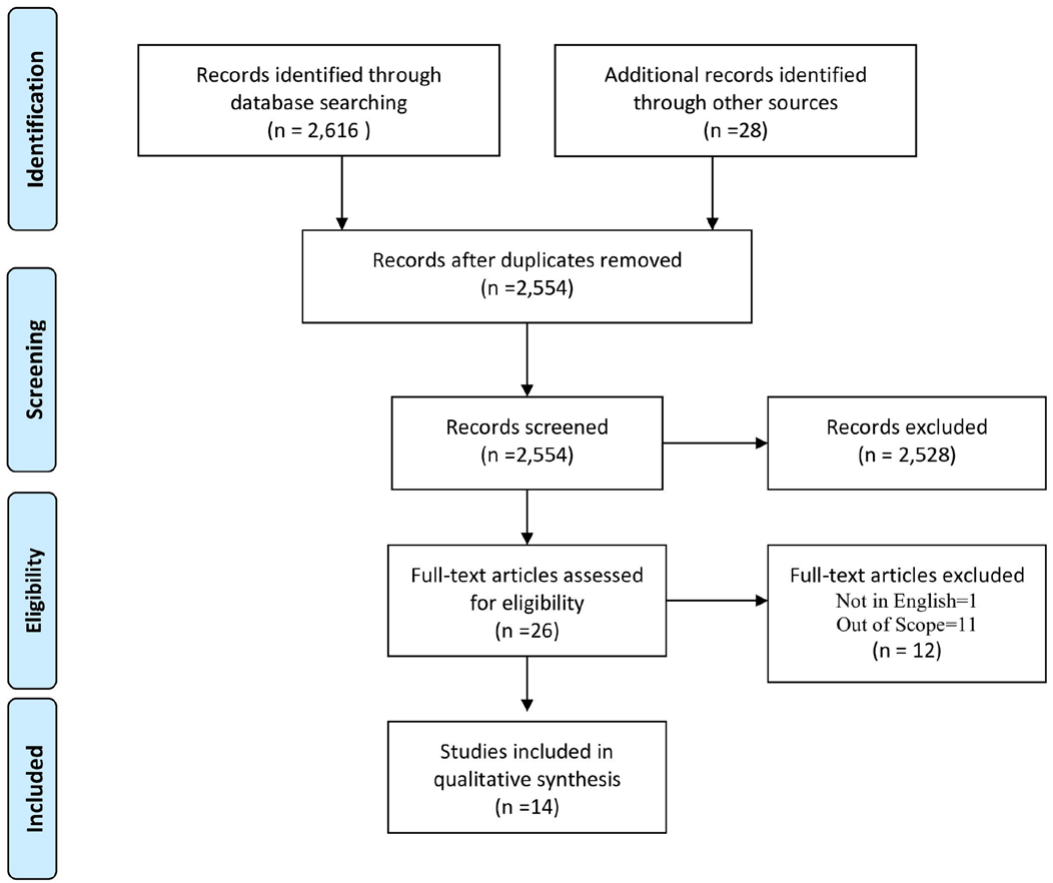
PRISMA Flowchart of the Study.

The initial search strategy identified 2644 studies. After the removal of 90 duplicate articles, the titles of 2554 studies were screened, and a further 2528 were excluded as being out of scope, leaving 26 articles for full-text evaluation. Of the 26 articles evaluated, only 14 met the eligibility criteria and were considered for this systematic review. The PRISMA selection process is illustrated in the accompanying Figure 1.

### Study Characteristics

The studies were published between 2018 and 2023. Five studies were conducted in the United States, 3 in France, 3 in the United Kingdom, and 1 each in Australia, Ireland, and Canada. Six studies^11-16^ compared the environmental impact of single-use and reusable urology devices using life cycle assessment, as depicted in Figure 1. Eight studies recommended some initiatives to reduce carbon footprint and energy consumption in urology practices.^5,8,17-22^ The summary of study characteristics is presented in Table 1.

### Life Cycle Assessment

**Figure 2.**
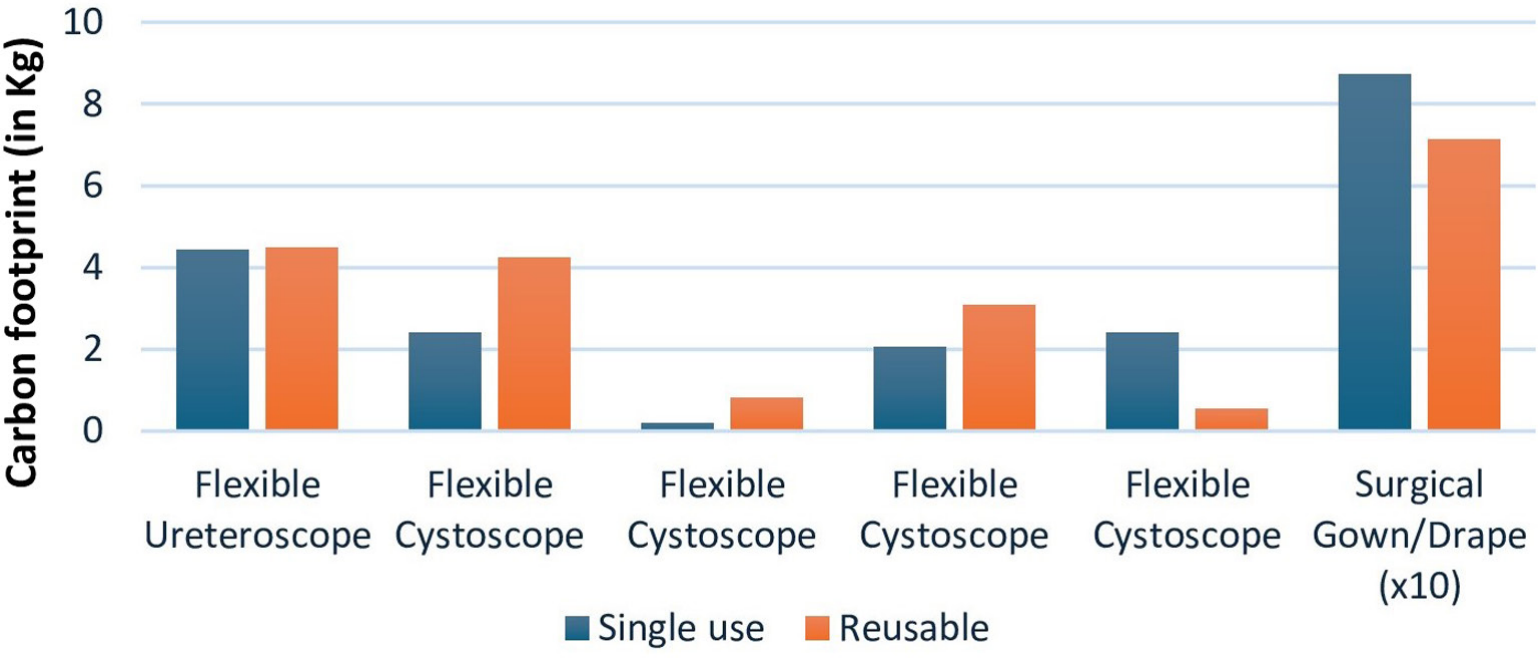
Carbon footprint of single-use and reusable urology instruments and consumables.

Of the 6 studies that carried out life cycle assessment of urology instruments, 4 studies focused on cystoscopes, 1 study focused on flexible ureteroscopes, and 1 focused on surgical consumables such as drapes and gowns. Whilst 3 of these studies concluded that single-use devices were more eco-friendly, 2 favored reusable options, and the final study found no discernible difference. Figure 2 compares the carbon footprint of single-use and reusable urology instruments and consumables.

The reported studies on cystoscopes and ureteroscopes used Cysto-Nephro Videoscope CYF-VA2 (Olympus) as the reusable device. This was compared with aScope 4 Cysto (Ambu), a single-use cystoscope, and LithoVue (Boston Scientific), a single-use ureteroscope. The major contributor to the environmental footprint of single-use devices is the waste generation from their disposal. Within the reusable group, the sterilization process was found to offset the expected benefit in some of the studies.

### Recommended Initiatives to Reduce Carbon Footprint

Eight studies recommended some initiatives to reduce carbon footprint and energy consumption in urology practices.^5,8,17-22^ The recommendations cut across several steps in the delivery of urological care to patients. These initiatives are presented in Table 2.

## Discussion and Conclusion

### Environmental Impact of Single-use and Reusable Urology Devices

The environmental impact of urology devices, whether single-use or reusable, can be assessed through a life cycle assessment (LCA) that considers various stages from raw material extraction to disposal. We found 6 studies that compared the environmental impact of single-use and reusable urology devices using life cycle assessment (Figure 2).

The cystoscope is the most studied urologic device used in 4 studies.^12-15^ Three of the 4 studies^12,13,15^ concluded that reusable cystoscopes have a greater environmental carbon footprint than single-use ones, while the last study^14^ reported that reusable cystoscopes have a lesser carbon footprint than single use. This could be explained by the enormous amount of energy consumption and carbon dioxide (CO_2_) production from the sterilization process and repackaging.^13,14^

The strict regulations around the process of reusable equipment are outlined in the European Union (EU)’s framework called the “Commission Implementing Regulation (EU) 2020/1207.”^23^ This regulation, operating under the broader “Regulation (EU) 2017/745,” establishes stringent requirements to ensure that reprocessed single-use devices meet the same safety and performance standards as their original counterparts. It emphasizes the need for comprehensive risk assessments, rigorous validation of reprocessing procedures, and thorough traceability of reprocessed devices. These requirements are designed not only to safeguard patients but also to potentially extend the life cycle of medical devices that might otherwise contribute to environmental waste.^23^

The remaining studies^11,16^ focused on ureteroscopes and surgical drapes. There was no significant difference in the carbon footprint of reusable and single-use ureteroscopes as the benefit of reusable devices was canceled out by the enormous energy and carbon dioxide generation from the sterilization process.^11^ The carbon footprint was found to be lower for reusable surgical drapes and gowns. The use of reusable surgical drapes and gowns in place of disposable ones was found to reduce CO_2_ emission by 18.2%.^16^

It is important to acknowledge that both reusable and single-use endoscopes make significant contributions to the carbon footprint. Some of the studies reviewed have revealed that the sterilization process can offset the potential benefits of reusable devices, underscoring the importance of selecting a sterilization method with minimal carbon or energy consumption while still maintaining effective infection prevention measures.^14^

### Initiatives and Practices in Urology that Contribute to Sustainability

Urology practices produce enormous clinical waste, thereby contributing significantly to the environmental footprint. Some recommended initiatives and practices that can contribute to sustainability in urology are summarized in Table 2.

### Surgeon Led Initiatives

Surgeon-led initiatives may include encouraging the manufacturing and use of all urology instruments with greater environmental considerations; streamlining instrument trays; editing preference cards to remove unused items; and shifting from disposable to reusable items to decrease waste and GHG production and reduce costs.^5^ Urologists ought to contemplate the environmental ramifications of clinical practices, such as employing single-use catheters for self-intermittent catheterization, particularly since this practice has not demonstrated superiority in preventing urinary tract infections (UTIs) over reusable catheters.^19^

Other initiatives which have been found to have noteworthy sustainability impact are the adoption of one-stop clinics and centralizing urology services. Both measures help in minimizing patients’ travel, lowering environmental impact, and making healthcare more sustainable overall.^24^

Adopting barcodes and quick response codes (QR codes) in urology can enhance sustainability by improving resource efficiency, reducing medical errors, saving time, and minimizing waste. This leads to a more efficient, cost-effective, and environmentally friendly urological practice.^26^ In a study, the extrapolated annual estimates place the CO_2_ emissions from printing one specific (Transurethral Resection of the Prostate) patient information leaflet multiple times at almost 4 kg for a single consultant’s practice.^26^ This figure can be easily minimized through the adoption of QR codes.

Day case procedures such as day case transurethral resection of bladder tumor (TURBT), which is standard practice for selected patient groups across multiple NHS hospitals, reduce the environmental footprint. The NHS achieved a carbon footprint reduction of 24 kg per patient over 8 years due to an increase in day-case TURBT; this is enough to power 2716 UK homes for a year.^17^

### Reducing Low-Value Clinical Care

Prostate MRI and biopsy procedures have a notable environmental footprint. A life cycle assessment study revealed that a single prostate biopsy, inclusive of prior prostate MRI, targeted and systematic biopsies, and subsequent pathology analysis, generates approximately 80.7 kg CO_2_e emissions. The MRI itself is the largest contributor, accounting for an estimated 42.7 kg CO_2_e (52.9% of the total emissions), primarily due to the significant energy consumption required for the procedure. The prostate biopsy procedure on the other hand contributes 33.3 kg CO_2_e, while pathology analysis adds an additional 4.8 kg CO_2_e.^18^

Performing systematic biopsies without the preceding MRI in some selected patients can significantly reduce emissions, resulting in a reduction of 4.5 metric tons of CO_2_e per 100 000 biopsies.^18^ This highlights the importance of considering environmental sustainability in medical procedures and the potential benefits of optimizing clinical pathways to reduce carbon emissions.

### Exploring the Possibility of a Drape-Free Procedure

An audit on the impact of drapes routinely used for cystoscopy on the environment showed that patient drapes generated an average of 295 g of plastic waste, about 26% of case waste by weight. Annually, this amounts to 1252.9 kg of waste generated by drapes alone and costs US$4969 per year.^20^ The role of personal protective equipment (PPE) and drapes in the prevention of post-cystoscopy UTI is still under debate, with studies describing UTI incidence between 1% and 21%.^27^ Given the impact of plastic drapes on the environment, it is worth looking into the possibility of drapeless cystoscopy in carefully selected patients.

### Ecological Impact of Innovative Procedures in Urology

Urology has been experiencing a lot of innovation and advancement in diagnostic and minimally invasive treatment due to the increasing incidence of urological diseases such as kidney stones, urinary tract conditions, and cancers. The resultant expansion of urological devices has brought about a significant decrease in morbidity, as well as the provision of aesthetic benefits.^21^ However, these benefits occur at a cost to the environment. For instance, the total carbon footprint of a robotic-assisted procedure was estimated to be 77% greater than that of open surgery (40.3 kg CO_2_-eq vs 22.7 kg CO_2_-eq/ patient).^28^ We must therefore consider interventions to mitigate this development to balance the danger posed by such procedures on the environment.

### Adoption of Fluid Management System

About a third of operating room surgical waste is fluid, and a large proportion of this fluid is produced in urology theaters. The disposal of this liquid waste by collection in plastic canisters, solidification, and transport for incineration or landfill is energy-intensive and ecologically unfriendly. The use of fluid management systems such as Neptune 3 (Stryker, Kalamazoo, Michigan, USA) prevents the use of disposable plastic and allows drainage of all urology irrigation fluid into the standard drain. A study on the environmental impact of fluid waste disposal in urology revealed that about 3203 kg CO_2_ production and 1114 plastic suction canisters can be saved over 8 months if the Neptune fluid waste management system is used.^22^

### Eco-friendly Anaesthesia

Inhaled anesthetic agents account for 0.01%-0.10% of total global carbon dioxide equivalent (CO_2_e) emissions, 5% of acute hospital CO_2_ emissions, and 50% of perioperative department emissions in high-income countries. From an anesthetics perspective, practitioners are encouraged to increase the use of spinal anesthesia for procedures where it is a feasible and achievable alternative to general anesthesia, such as nephrolithotomy and transurethral resection of the prostate and bladder tumors. When general anesthesia is a must, propofol-based intravenous anesthesia should be considered. The use of local anesthesia for procedures like cystoscopy should also be encouraged.^8^

### Telemedicine and Carbon Footprint

Telemedicine is the use of information and communications technologies (ICT) to aid healthcare delivery. Telemedicine offers significant potential for reducing the carbon footprint associated with healthcare through the reduction in transportation frequency.^28^ Transportation accounts for approximately 7% of the total carbon footprint of healthcare and 10% of the NHS’s greenhouse gas (GHG) emissions. In 2008, around 5% of all road travel in the UK was attributable to the NHS. By reducing the need for travel, telemedicine can play a crucial role in mitigating the healthcare sector’s impact on climate change.^29^

Within Urology, examples of such telehealth initiatives include virtual urology clinics and virtual ward rounds. A systematic review of adult urology telehealth or virtual clinic strategies unveiled an annual carbon footprint reduction ranging from 0.7 to 4.35 metric tonnes of CO_2_ emissions.^30^

Healthcare significantly contributes to global carbon emissions and pollution, and it requires a more comprehensive approach to sustainability beyond the endorsement of reusable equipment. A holistic strategy must address all stages of the lifecycle, from manufacturing and sterilization to proper disposal, to effectively reduce the carbon footprint. Important elements to consider include the promotion of low-emission anesthetics and the integration of advanced technologies such as barcoding for medical documentation and telehealth initiatives like virtual urology clinics and virtual ward rounds. These measures collectively play a pivotal role in minimizing emissions and advancing sustainable practices in healthcare. By implementing such multifaceted strategies, the healthcare sector can make significant strides toward reducing its environmental impact.

## Figures and Tables

**Table 1.** Study Characteristics

Author	Country of Origin	Journal	Objective of Study	Main Findings/Recommendation
Davis et al, 2018^11^	Australia	*Journal of Endourology*	Comparison of the environmental impact of single-use versus reusable flexible ureteroscopes through life cycle analysis.	The environmental impacts of reusable and single-use flexible ureteroscopes are comparable. The single-use LithoVue flexible ureteroscope generates 4.43 kg of CO_2_ per procedure, while the Olympus reusable ureteroscope has a carbon footprint of 4.47 kg of CO_2_ per procedure.
Hogan et al, 2022^12^	Ireland	*Journal of Endourology*	To compare the carbon footprint of single-use vs reusable flexible cystoscopes through life cycle 14.	Disposable flexible cystoscopes have a significantly lower impact on the environment than reusable cystoscopes in terms of carbon footprint. A total of 2.41 kg of CO_2_ was produced per case for the single-use flexible cystoscope, compared with 4.23 kg of CO_2_ for the reusable cystoscope.
Boucheron et al, 2022^13^	France	*Journal of Endourology*	To quantify the environmental impact and costs associated with flexible cystoscopy procedures. Comparison between disposable and reusable cystoscopes.	Implementing a strategy of using 100% disposable cystoscopes was associated with similar costs and reduced waste generation and water consumption compared to reusable devices. The amount of waste generated by reprocessing reusable cystoscopes was 800 g per procedure, while reprocessing disposable cystoscopes generated 200 g.
Baboudjian et al^14^	France	*European Association of Urology*	Life cycle assessment of reusable and disposable flexible cystoscopes.	The carbon footprint of a single reusable flexible cystoscope was 3.08 kg CO_2_ per procedure, while that of a disposable flexible cystoscope was 2.06 kg CO_2_ per procedure. Sterilization of reusable cystoscopes was found to have a significantly larger environmental footprint and impact than the lifespan of the single-use cystoscope.
Kemble et al, 2022^15^	USA	*British Journal of Urology International*	To compare the carbon footprint and environmental impact of single-use and reusable flexible cystoscopes.	The environmental impact of reusable flexible cystoscopes is markedly less than single-use cystoscopes (2.40 kg versus 0.53 kg per case). The total estimated per-case carbon footprint of single-use devices was 2.40 kg CO_2_, while that of reusable devices was 0.53 kg CO_2_, favoring reusable devices.
Tsang et al, 2022^16^	UK	*Journal of Clinical Urology*	Comparing carbon footprints of disposable and reusable items in urology procedure.	In 1 month, using disposable drapes and gowns for 648 urology procedures generates 56 482 kg of waste (87.16 kg per procedure). Substituting disposable items with reusable surgical drapes and gowns would reduce CO_2_ emissions by 18.2%, resulting in a total of 46 203 kg of waste (71.30 kg per procedure).
Kornberg et al, 2023^5^	USA	*European Association of Urology*	To highlight areas of interest in urology, as well as opportunities for surgeon-led initiatives to reduce the energy and waste footprint of urologic care.	Surgeon-led initiatives include: streamlining instrument trays, editing preference cards to remove unused items, and shifting from disposable to reusable items, which have the potential to decrease waste and GHG emissions. All endoscopes should be manufactured with greater environmental considerations. Urologists should consider the environmental impacts of clinical practices such as intermittent catheterization.
Phull et al, 2023^17^	UK	*European Association of Urology*	To investigate the difference in the carbon footprint between day case and inpatient transurethral resection of bladder tumor (TURBT) surgery in England.	The study examined 209 269 patients that underwent TURBT procedures across all NHS hospitals in England between 2013 and 2022. On average, 41 583 (20%) were day cases, and 167 686 (80%) were inpatient cases. The rate of day case TURBTs increased progressively from 13% in 2013 to 31% in 2022, resulting in an estimated cumulative carbon saving of 2.9 million kg CO_2_e over the study period. This saving is equivalent to powering 2716 UK homes with electricity for 1 year.
Leapman et al, 2023^18^	USA	*European Association of Urology*	To estimate the environmental impacts associated with prostate magnetic resonance imaging (MRI) and prostate biopsy.	In the USA, a single transrectal prostate biopsy, including prostate MRI and targeted and systematic biopsies, emits an estimated 80.7 kg CO_2_e. MRI alone contributes 42.7 kg (52.9%) of this emission. Performing systematic biopsies without the preceding MRI in selected patients could reduce emissions by 4.5 metric tons of CO_2_e per 100 000 biopsies. The use of MRI as a triage strategy to select biopsy candidates and limit sampling to MRI-evident areas could reduce emissions by 1.4 million kg CO_2_e per 100 000 patients.
Elliott et al, 2023^19^	USA	*European Urology Focus*	Single use versus reusable catheters for intermittent catheterization.	Eight different randomized trials with the largest sample of 80 patients compared symptomatic UTI rates between users of single-use and reusable catheters over up to 1 year of follow-up with no difference in symptomatic UTI rates observed. To enhance healthcare sustainability, transitioning from single use to reusable catheters for intermittent catheterization is recommended.
Belliveau et al, 2023^20^	Canada	*European Association of Urology*	A single-centre prospective audit of cystoscopy waste at a tertiary hospital to quantify the contribution of drapes to overall cystoscopy waste.	Cystoscopy drapes offer a target for waste reduction in urological practice. Patients’ drapes contribute an average of 295 g (26%) to the total 1125 g of waste produced during a single cystoscopy procedure. Annually, patient drapes account for 1252.9 kg of the total 4777.7 kg of waste generated from cystoscopy procedures, amounting to a cost of US$4969 per year.
Misrai et al, 2020^21^	France	*European Association of Urology*	To review the Carbon Footprint of New Minimally Invasive Surgical Technologies in Urology.	The increasing demand for advanced diagnostic and minimally invasive treatments in urology benefits patients but comes at an environmental cost. For example, the total carbon footprint of robotic-assisted laparoscopy (40.3 kg CO_2_-eq per patient) was estimated to be 77% higher than that of open surgery (22.7 kg CO_2_-eq per patient).
Pandit et al,2023^8^	USA	*European Urology Focus*	To highlight actionable measures and ideas that can lead to greener, healthier, and more sustainable urological practice.	Modification of autoclaving to generate sterile water. Conscious use of disposable and reusable instruments is advised. Anesthetic agents, including potent greenhouse gases like nitrous oxide and desflurane, contribute 0.01%-0.10% to global CO_2_e emissions. They make up 5% of acute hospital emissions and 50% of perioperative department emissions in high-income countries. Encouraging the adoption of spinal, intravenous, and local anesthetics can help reduce their environmental impact.
Gunner et al, 2022^22^	UK	*Journal of Clinical Urology*	Comparing fluid, CO_2_ and cost savings of Neptune fluid waste management system with traditional incineration or landfill disposal.	The Neptune fluid waste management system saves the equivalent of 3203 kg of CO_2_ in fluid waste, prevents the production of 1114 plastic suction canisters, and avoids the use of 290 lengths of plastic tubing. This system not only reduces costs but also saves theater staff time.

GHG, greenhouse gas; MRI, magnetic resonance imaging; NHS, National Health Service; TURBT, transurethral resection of bladder tumor; UTI, urinary tract infection.

**Table 2.** Initiatives to Reduce Waste Footprint and Energy Consumption in Urology Practices

Focus Areas	Recommended Initiatives
Surgeon-led initiatives	Increasing the frequency of day case procedures, minimizing low-value clinical care, such as unnecessary prostate biopsies by utilizing MRI, using quick response (QR) codes in documentation, centralizing services in one-stop clinics, telemedicine
Drapes	Exploring drape free cystoscopy for specific patients
Fluid waste	Implementing fluid management systems to conserve water
Anaesthesia	Promoting the use of local, spinal, and propofol-based anaesthetics
Instruments trays	Optimizing instrument trays, updating preference cards to eliminate unnecessary items, and transitioning from disposable to reusable items
GHG Emission Inventory	Establishing a precise inventory of both direct and indirect greenhouse gas (GHG) emissions for urology procedures

GHG, greenhouse gas; MRI, magnetic resonance imaging.
